# Health and Dietary

**Published:** 1905-07-01

**Authors:** 


Vol. XXXVIII.—No. 979. SATURDAY, JULY 1, 1905.

### Health and Dietary.

It is very satisfactory to see, in the columns of many of our lay contemporaries, that a large amount of public attention is being directed towards questions of dietary, and that, notwithstanding the prevalence of many half-truths and of more exaggerations, there is evidence of a manifest desire on the part of the present generation to live more wisely than was the custom of their ancestors. The novels of Charles Dickens may be regarded as furnishing the most authentic record in our possession of the manners and customs of our parents and grandparents; and, throughout nearly the whole of them, the general tendency to over-eating is almost as pronounced as the tendency to free drinking. It seems to have been regarded as distinctly meritorious to consume huge quantities of animal food; and the characters by whom this form of merit was most conspicuously displayed were at the same time conspicuous for all the virtues with which writers of fiction are privileged to endow their creations. The good people of the Dickens series not only eat, but they fatten upon their eating; while the unprincipled or the base, the Jingles and the Uriah Heeps, are almost invariably lean. It was not so much that these failed to eat, whenever they had favourable opportunities, but that food failed to nourish them after the fashion in which it nourished the jovial and the benevolent. The elements of happiness, as described by the French philosopher, were divorced from one another, and the bad heart was no longer attended by the good digestion. The most popular prose fiction of a nation is, at least, as powerful in governing conduct as its ballads, and romance exerts a far wider influence than physiology. Romance represented Mr. Pickwick and Mr. Wardle as types of the most praiseworthy forms of human activity; physiology, as personified by a physician, would probably have rejected them as applicants for life insurance. The increased longevity of the last half-century is probably due as much to diminished eating as to any other of the many single causes to which it has been attributed.

It is curious to remember, in relation to food, how little the human race has been governed by the teachings of experience, and how much, in many successive stages of history, by something which might perhaps be described as fashion.

Even from very remote times, however, there has been no lack of evidence that a restricted diet is not incompatible with, or is even conducive to, a very high degree of health, of vigour, and of longevity. Between the Israelitish conquerors of Canaan, "who could live on a few dates soaked in butter and a mouthful of milk a day" and to whom "rapine was a business and massacre a sport," and the Scotch of the beginning of the last century, who "cultivated literature upon a little oatmeal," there were many parallel instances, all alike exhibiting evidence that a meagre diet had in no way detracted from their physical or mental powers. In the beginning of the sixteenth century Luigi Cornaro, then a man of forty whose health had become impaired, placed himself upon the regimen which his writings have rendered historical, and of which the last account, revised by his own hand, was published fifty-five years later. He commenced by limiting himself to a daily allowance of twelve ounces of solid food and fourteen ounces of wine, but soon found it possible to effect a still further diminution, and at the age of seventy is said to have suffered severely from having been betrayed into an excess to the amount of two ounces beyond his customary quantity. Few things are more remarkable than the slenderness of the following which he obtained, notwithstanding the wide interest which his experience and his publications seem to have excited among his contemporaries; but the fact is that over-eating may be a vice so agreeable in itself, and, when practised in what may be described as moderation, one so slightly attended by any immediately recognisable ill-consequences, that many of those who yield to it would emphatically deny the true character of their action, and would eagerly assert that their appetites were strictly regulated by the demands of their bodies. The fact remains that many of the ailments of two different kinds of people, namely, the wealthy or merely prosperous middle classes, and their servants, are well known to the medical profession to be largely due to the habitual over-eating which is practised without thought and merely as a matter of custom. There is an old story of an Irishman who gave an acceptance to an urgent creditor, and exclaimed "thank God, that's done with." The man who every day partakes of *hors d'œuvres*, soup, fish, *entrées*, joint, game or some substitute for it, sweets, and dessert, has likewise given an acceptance, and will certainly some day be called upon to honour it on demand. The vagaries of vegetarianism or other fanciful plans of diet resolve themselves into this, that it is essential to health to eat moderately, to masticate food thoroughly, and not to eat too much. If these rules be observed, the character of what is eaten may be regarded as a matter of comparatively small importance.

